# Using short knee radiographs to predict the coronal alignment after TKA: Is it an accurate proxy for HKA on full-length images?

**DOI:** 10.1186/s13018-022-03235-w

**Published:** 2022-07-06

**Authors:** Guangqian Shang, Mingwei Hu, Jianjun Guo, Xu Hao, Shuai Xiang

**Affiliations:** Department of Joint Surgery, The Affiliated Hospital of Qingdao University, No. 59, Haier Road, Qingdao, 266000 Shandong China

**Keywords:** Coronal alignment, Total knee arthroplasty, Hip-knee-ankle angle, Femorotibial angle

## Abstract

**Background:**

The postoperative clinical outcomes has been extensively demonstrated to correlate with the coronal alignment after total knee arthroplasty (TKA). However, in different studies, either the hip-knee-ankle angle (HKA) on a full-length radiograph or the femorotibial angle (FTA) on a short knee film was used to categorize the postoperative coronal alignment. Meanwhile, several different FTA ranges were regarded as neutral alignment in different studies. As a result, it is still unknown that how FTA on short knee films and HKA related to each other. The FTA may be able to become an accurate proxy of HKA to predict the coronal alignment. The purpose of this study was to explore the correlation between the FTA and the HKA after TKA and to find the most accurate FTA range.

**Methods:**

About 223 patients were included in this study and standard weight-bearing short knee films as well as full-length radiographs were acquired. The pre- and postoperative FTA, as well as the postoperative anatomical lateral distal femoral angle (aLDFA) and anatomical medial proximal tibial angle (aMPTA) were measured on short knee films by two orthopedic surgeons independently. On full-length films, the pre- and postoperative FTA, the pre- and postoperative HKA, as well as the postoperative mechanical lateral distal femoral angle (mLDFA) and mechanical medial proximal tibial angle (mMPTA) were also recorded by two other surgeons independently. Pearson correlation analysis was performed to compare FTA and HKA, aMTPA and mMTPA, aLDFA and mLDFA, respectively.

**Results:**

The postoperative FTA and HKA had a good correlation (*r* = 0.86). The agreements were reached 82.7%, 71.0%, and 68.2% of all patients using three previously reported FTA ranges. When analyzing the independent alignment of the tibial tray and the femoral component, 84.1% and 57.9% of all patients was reached an agreement on the classification.

**Conclusions:**

On most occasions, the consistence between the FTA and HKA in assessing the coronal limb alignment of the lower extremity and the tibial component is satisfactory. However, the postoperative full-length film is still needed to evaluate accurately the coronal alignment of the femoral component.

## Background

The coronal limb alignment is of importance in total knee arthroplasty (TKA). Although controversial, restoration of the neutral mechanical alignment of lower extremity has been traditionally regarded as one of the primary goals of TKA and is correlated with increased implant survivorship as well as functional outcomes in most previous studies [1–3]. Generally, the hip-knee-ankle angle (HKA) measured on a standing anterior–posterior (AP) full-length radiograph has been widely accepted as a standard measurement of the mechanical alignment of lower extremity. The post-operative HKA within 3° deviation of 0° is regarded as “neutral” when evaluating the position of the knee implants, and valgus positioning of the knee implant is commonly assigned a negative value [3, 4].

In the circumstances that the standing full-length radiographs are not available, the femorotibial angle (FTA) measured on a standing AP short knee film is used to indirectly predict the coronal alignment of lower extremity [5, 6]. The relationship between FTA and HKA is still controversial. Although there have been investigations reporting a linear relationship between preoperative FTA and HKA, other studies have concluded that the FTA on short knee films hampers the accurate classification of the alignment [7–9]. The investigation on the relationship between the postoperative FTA and HKA is even fewer and as a result, their relationship is beyond understanding. In previous studies, three postoperative FTA ranges have been regarded as “neutral alignment”. Ritter et al. reported a neutral alignment when the postoperative FTA was between 2.4° and 7.2°, whereas Kim et al. regarded the FTA within 3°–7.5° as neutral [10, 11]. A third criteria is proposed by Morgan et al., who considered a 4°–9° FTA as neutral in assessing the coronal alignment [12]. However, there lacks the commonly accepted FTA cutoff values to distinguish neutral alignment from varus, as well as valgus, and their accuracy in predicting the coronal alignment has been rarely studied.

The purpose of this study was to evaluate the correlation between the FTA measured on a standing short knee film and the HKA measured on a standing full-length radiograph after TKA and to find accurate FTA ranges when evaluating the postoperative coronal alignment. This hypothesis was that we could use the FTA as a proxy of HKA to predict the coronal alignment.

## Materials and methods

This study was approved by the ethics committee of our institution and written consent was obtained from all participants included in this study. A total of 223 patients were recruited from august 2018 to September 2019. The inclusion criteria were patients who received a unilateral TKA due to osteoarthritis or inflammatory arthritis. The exclusion criteria included: (1) patients with standing difficulties; (2) trauma history of the ipsilateral lower extremity; (3) patients who received a simultaneous bilateral TKA. Demographic information was recorded after recruiting.

All participants in this study received a TKA following the standard procedure, including an extra-medullary guided osteotomy of the distal femur with 6° valgus with respect to the femoral anatomical axis (FAA) and an intra-medullary guided osteotomy of the tibial plateau. A bone-cemented prosthesis with either posterior stable design or a medial-pivotal design was implanted.

Standing short AP knee films and weight-bearing full-length AP images were taken preoperatively as well as 3 to 6 months postoperatively for all participants. A senior orthopedic resident specifically guided the patients to guarantee the full extension of the knee and the forward-facing of the patella during film shooting.

**Fig. 1:**
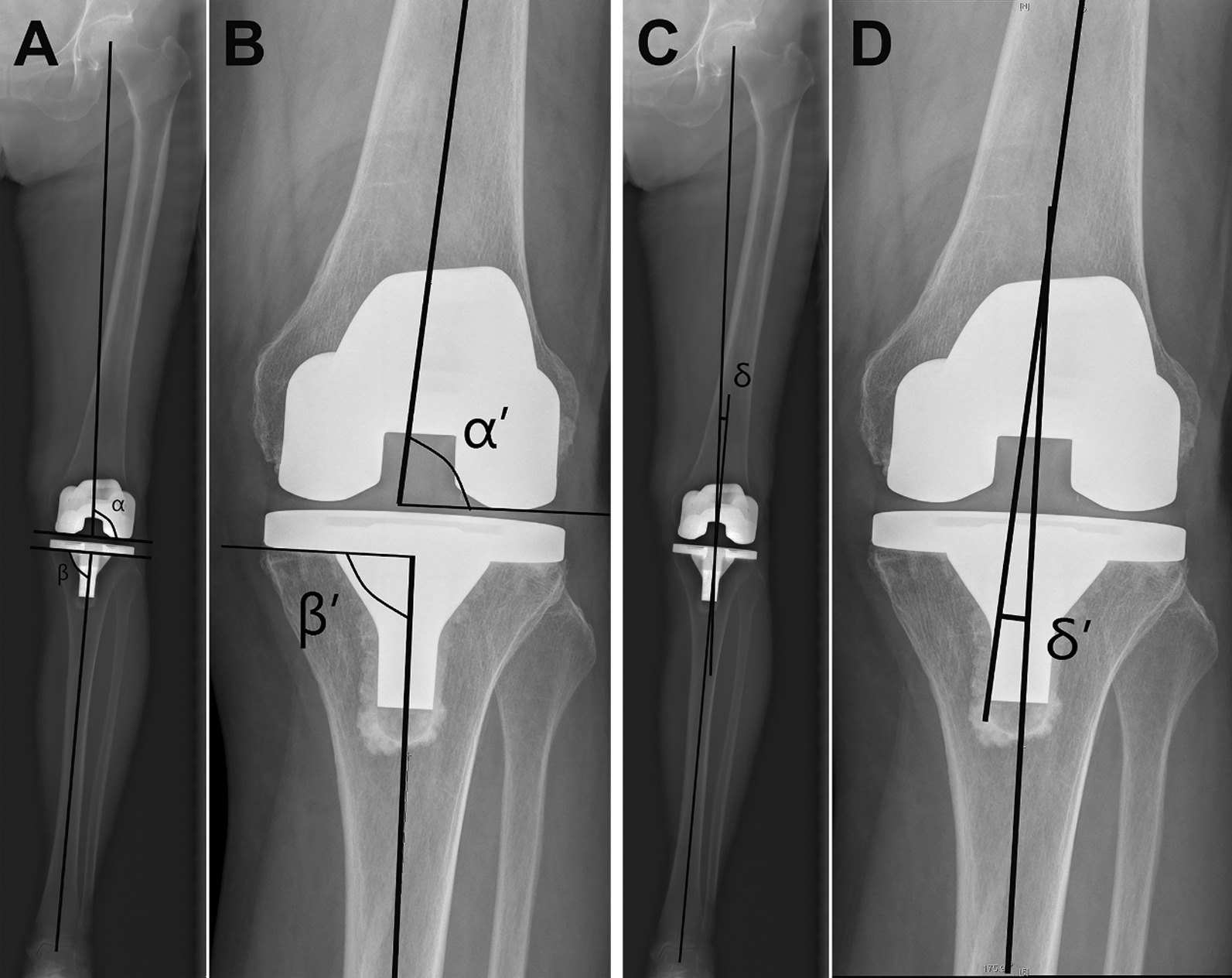
The measurement of the investigated angles on short knee films and full-length films in this study. **A** α: mMTPA, β: mLDFA. **B** α’: aMTPA, β’: aLDFA. **C** δ: HKA. **D** δ’: FTA. mMPTA, mechanical medial proximal tibial angle; mLDFA, mechanical lateral distal femoral angle; aMPTA, anatomical medial proximal tibial angle; aLDFA, anatomical lateral distal femoral angle; HKA, hip-knee-ankle angle; FTA, femorotibial angle

The images were read on a PACS (General Electric, Chicago, IL, USA) monitor (2 K × 2 K, 12bit) and measured using a mouse-point cursor and an automated computer calculation. Preoperatively, the FTA was measured on short knee films, and the HKA on full-length AP images (Fig. 1). Postoperatively, the FTA, anatomical medial proximal tibial angle (aMPTA), and anatomical lateral distal femoral angle (aLDFA) were measured on short knee films. On full-length AP images, the HKA, mechanical lateral distal femoral angle (mLDFA), and mechanical medial proximal tibial angle (mMPTA) were measured (Fig. 1). All measurements were done using the standard method reported by Park et al. [13].

The HKA value less than − 3° was considered valgus, neutral between − 3° and 3°, and varus greater than 3°. When evaluating the coronal alignment on short knee films, three FTA ranges reported by Ritter, Kim, and Morgan were employed, who regarded the FTA within 2.4° to 7.2°, 3° to 7.5°, and 4° to 9°, respectively as neutral postoperative alignment [10–12]. With the FTA value decreases, the coronal alignment become more varus and vice versa.

We used aMPTA on short knee films and mMPTA on full-length images to determine the alignment of tibial component. The criteria reported by Parratte et al. was employed to evaluating the varus, neutral, or valgus of tibial component. Briefly, the tibial component was considered as varus when the aMPTA or mMPTA was less than 88°, neutral when the aMPTA or mMPTA was between 88° and 92°, and valgus when the aMPTA or mMPTA was greater than 92° [3]. The coronal alignment of the femoral component was determined by aLDFA on short knee films and mLDFA on full-length images. On short knee films, the femoral component was considered as neutrally aligned when aLDFA-s ranged from 82° to 86°, valgus when aLDFA < 82°, and varus when aLDFA > 86°. On full-length images, the femoral component was considered as neutrally aligned when mLDFA-s ranged from 88° to 92°, valgus when mLDFA < 88°, and varus when mLDFA > 92° [14].

Two orthopedic surgeons measured the full-length images independently using the software-provided protractor. If consensus was reached on the classification of the alignment, then the averaged value was used. However, if discordant classification was assigned, a third measurement was done by the two surgeons together to determine the final classification. Two other surgeons blinded to the results of the full-length films measured the short knee films and same method was used to get the results.

Statistical analyses were performed using Prism 7 software (GraphPad Software, San Diego, CA, USA). Intra- and inter-class correlation coefficients (ICC) with 95% confidence interval (CI) were used to assess intra- and inter-observer variability. The Kolmogorov–Smirnov test was used to determine the normality of the data. Continuous variables are expressed as means and standard deviations (SDs). Pearson correlation analysis was performed to compare FTA and HKA, aMTPA and mMTPA, aLDFA and mLDFA, respectively. To evaluate the correlation coefficients, the criteria reported by Park et al. [13]. was employed. Briefly, 0.9 ≤ *r* ≤ 1 was excellent, 0.7 ≤ *r* < 0.9 was good, 0.5 ≤ *r* < 0.7 was fair, 0.25 ≤ *r* < 0.5 was low, and *r* < 0.25 was poor. According to different FTA ranges, the agreement rates between FTA and HKA were performed using Fisher's exact test. *P* < 0.05 indicated statistical significance.

## Results

Preoperative and postoperative alignment of the patients in this study is listed in Table 1. The preoperative coronal alignment was determined by preoperative HKA. Good to excellent intra- and inter-observer variability was achieved for all postoperative measurements with an inter-rater ICC between 0.873 and 0.944 and an intra-rater ICC between 0.874 and 0.954 (Table 2).

**Table 1:** Preoperative and postoperative alignment of the 223 knees determined by on full-length films

Parameters	Preoperative	Postoperative
Coronal alignment		
Neutral (−3° ≤ HKA ≤ 3°)	18 (8.1%)	83 (37.2%)
Varus (HKA > 3°)	200 (89.7%)	126 (56.5%)
Valgus (HKA < −3°)	5 (2.2%)	14 (6.3%)
Alignment of the tibial tray		
Neutral (88° ≤ mMPTA ≤ 92°)		127 (56.9%)
Varus (mMPTA < 88°)		78 (35.0%)
Valgus (mMPTA > 92°)		18 (8.1%)
Alignment of the femoral component		
Neutral (88° ≤ mLDFA ≤ 92°)		104 (46.6%)
Varus (mLDFA > 92°)		100 (44.8%)
Valgus (mLDFA < 88°)		19 (8.6%)

HKA, hip-knee-ankle angle; mMPTA, mechanical medial proximal tibial angle; mLDFA, mechanical lateral distal femoral angle

**Table 2:** Intra- and inter-observer variability of postoperative FTA, HKA, aMPTA, mMPTA, aLDFA, mLDFA

	First versus second assessment by 1 observer		Assessment by observer 1 versus observer 2	
	ICC	95%CI	ICC	95% CI
FTA	0.873	0.845–0.894	0.906	0.869–0.922
HKA	0.932	0.907–0.951	0.954	0.941–0.966
aMPTA	0.915	0.883–0.928	0.874	0.862–0.895
mMPTA	0.910	0.876–0.922	0.927	0.904–0.938
aLDFA	0.893	0.858–0.914	0.884	0.862–0.903
mLDFA	0.944	0.920–0.956	0.936	0.921–0.958

*FTA* femorotibial angle, *HKA* hip-knee-ankle angle, *aMPTA* anatomical medial proximal tibial angle, *mMPTA* mechanical medial proximal tibial angle, *aLDFA* anatomical lateral distal femoral angle, *mLDFA* mechanical lateral distal femoral angle, *ICC* intraclass correlation coefficient, *CI* confidence interval

**Fig. 2:**
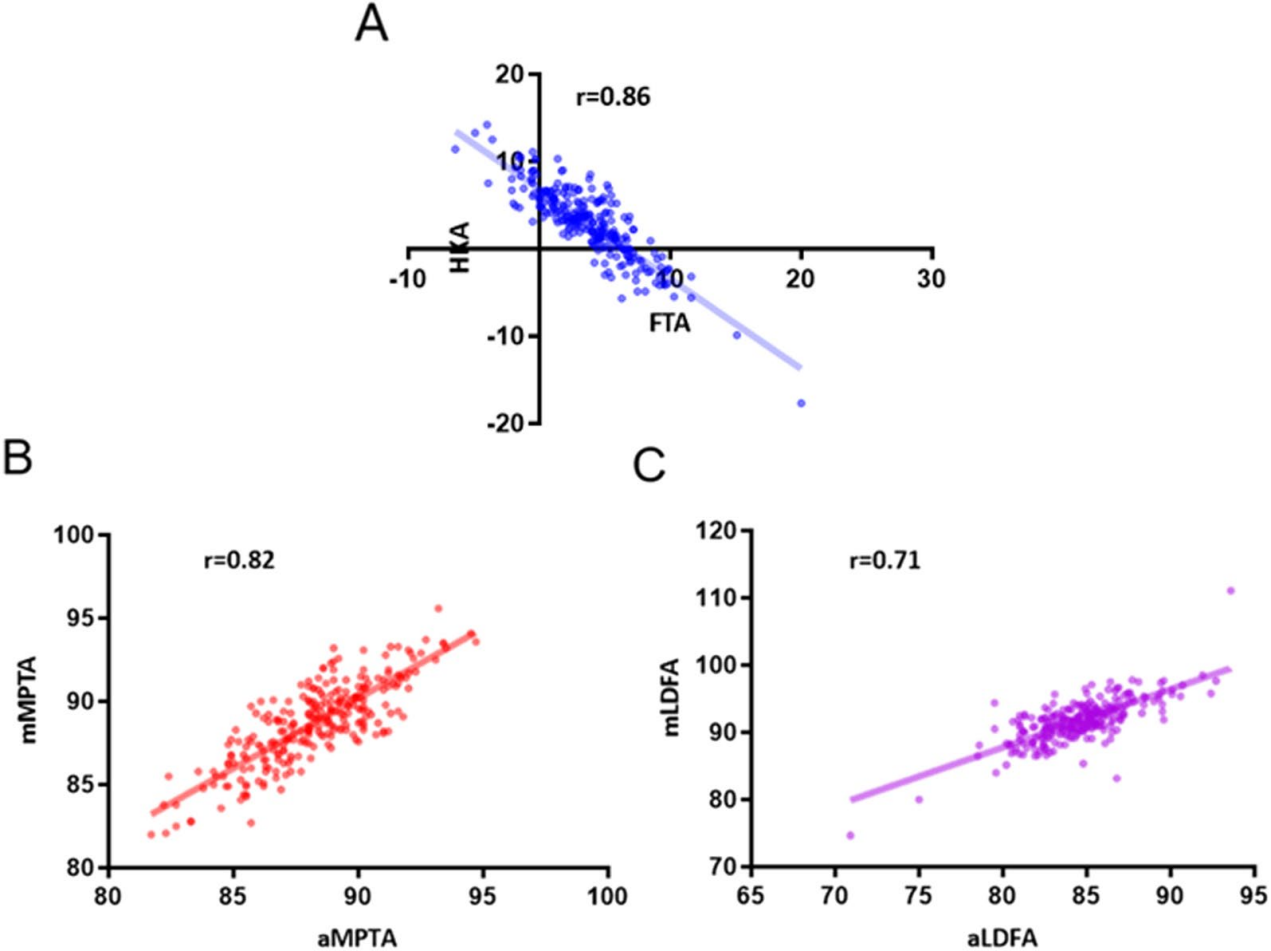
The correlation coefficient between postoperative angles measured on short knee films and full-length films. ° The FTA and HKA had a good correlation, with an r of 0.86. **B** The aMTPA and mMTPA had a good correlation, with an r of 0.82. **C** The aLDFA and mLDFA had a good correlation, with an r of 0.71. FTA, femorotibial angle; HKA, hip-knee-ankle angle; aMPTA, anatomical medial proximal tibial angle; mMPTA, mechanical medial proximal tibial angle; aLDFA, anatomical lateral distal femoral angle; mLDFA, mechanical lateral distal femoral angle

**Fig. 3:**
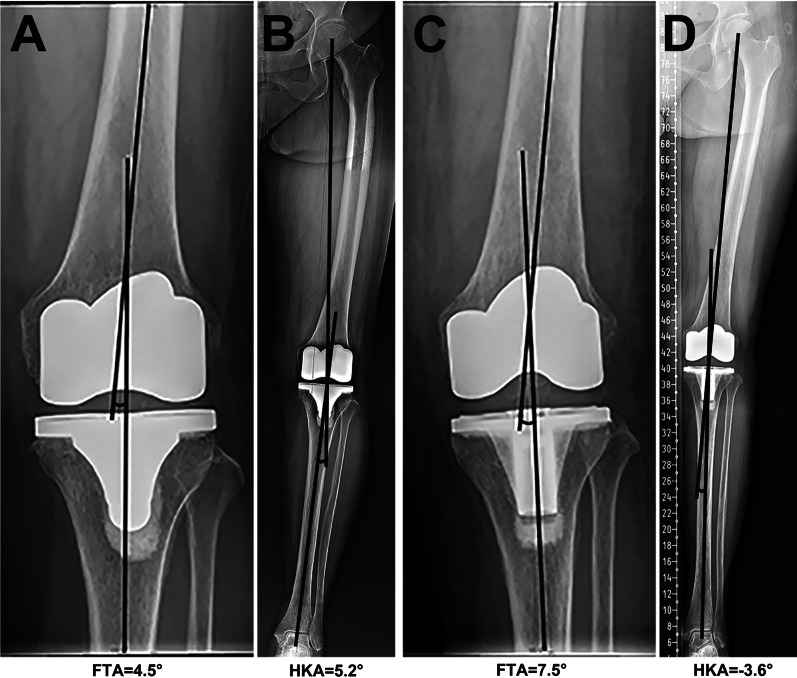
Cases with a neutral FTA while a varus or valgus HKA. **A** and **B** The FTA is neutral (within 4°–9°) but the HKA is varus (more than 3°). **C** and **D** The FTA is neutral but the HKA is valgus (less than −3°). FTA, femorotibial angle; HKA, hip-knee-ankle angle

**Fig. 4:**
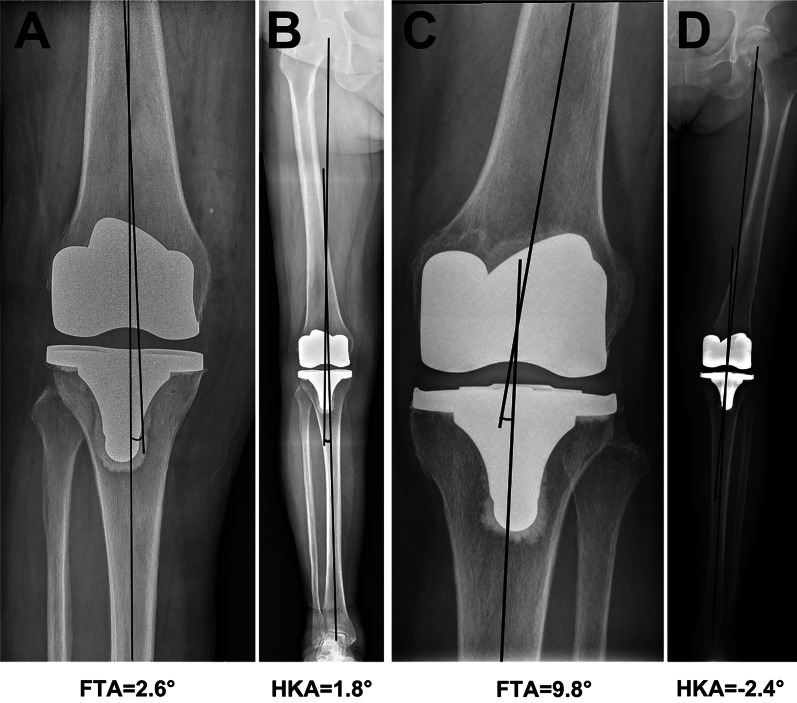
Cases with a neutral HKA while a varus or valgus HKA. **A** and **B** The HKA is neutral (within −3° to 3°) but the FTA is varus (less than 4°). **C** and **D** The HKA is neutral but the FTA is valgus (more than 9°). FTA, femorotibial angle; HKA, hip-knee-ankle angle

Overall, a good correlation coefficient was identified between FTA and HKA, with an *r* value of 0.86 (Fig. 2) and the equation between the FTA and HKA was HKA = −1.03*FTA + 6.83. According to our results, the optimal range of FTA to define “neutral alignment” was 3.7° to 9.5°, which was quite close to the criteria reported by Morgan et al. [12]. With our calculated FTA ranges, we identified 178 patients (79.8%) with agreements of the coronal alignment. We further examined the three existed FTA criteria reported by Morgan et al. [12], Kim et al. [11], and Ritter et al. [6] using our data. Detailed data are shown in Table 3. The FTA criteria reported by Morgan et al. was significantly more accurate than other criteria when predicting the classification of the coronal alignment. Despite the relatively high accuracy, there were 19 out of 126 patients (15.1%) who obtained a varus alignment while displaying a neutral FTA on short knee films, and 5 out of 14 valgus alignments (35.7%) showed a neutral FTA (Table 3 and Fig. 3). Meanwhile, 11 varus (13.3%) and 4 valgus (4.8%) FTA values were identified among 83 patients with neutral alignment (Table 3 and Fig. 4). The coronal alignment of the tibial component was also determined by mMPTA measured on full-length films. Detailed data of the alignment of tibial and femoral component on short knee films and full-length films was shown in Table 4. The mean mMPTA and aMPTA were 88.6° and 88.2°, respectively. The agreement of mMPTA and aMPTA was reached in 185 patients (83.0%) and a good correlation coefficient was identified, with an *r* value of 0.82 (Fig. 2). The agreement on the classification of the coronal alignment was reached on 133 patients (57.6%) when using aLDFA measured on short knee films to predict the alignment of the femoral component. And the correlation between mLDFA and aLDFA was lower than that between mMPTA and aMPTA, only 0.71 (Fig. 2).

**Table 3:** Classification of the coronal alignment based on HKA and FTA

HKA	Varus	Neutral	Valgus	Agreement rate (%)	*P* value
FTA calculated in this study					
Varus (FTA < 3.7°)	102	10	–	79.8	0.54
Neutral (3.7° ≤ FTA ≤ 9.5°)	24	70	8		
Valgus (FTA > 9.5°)	–	3	6		
FTA reported by Morgan					
Varus (FTA < 4°)	107	11	–	82.5	
Neutral (4° ≤ FTA ≤ 9°)	19	68	5		
Valgus (FTA > 9°)	–	4	9		
FTA reported by Kim					
Varus (FTA < 3°)	85	7	–	72.2	0.013
Neutral (3° ≤ FTA ≤ 7.5°)	41	65	3		
Valgus (FTA > 7.5°)	–	11	11		
FTA reported by Ritter					
Varus (FTA < 2.4°)	74	4	–	68.2	0.0006
Neutral (2.4° ≤ FTA ≤ 7.2°)	52	66	2		
Valgus (FTA > 7.2°)	–	12	12		

*HKA* hip-knee-ankle angle, *FTA* femorotibial angle

**Table 4:** Classification of the alignment of tibial and femoral component on short knee films and full-length films

Alignment on full-length film	Varus	Neutral	Valgus	Agreement rate (%)
aMPTA on short films				
Varus (aMPTA < 88°)	69	20	–	83.0
Neutral (88° ≤ aMPTA ≤ 92°)	9	106	8	
Valgus (aMPTA > 92°)	–	1	10	
aLDFA on short films				
Varus (aLDFA > 86°)	45	10	1	57.6
Neutral (82° ≤ aLDFA ≤ 86°)	50	77	7	
Valgus (aLDFA < 82°)	5	17	11	

*aMPTA* anatomical medial proximal tibial angle, *aLDFA* anatomical lateral distal femoral angle

## Discussion

Restoration of the neutral mechanical axis of the lower extremity has long been considered as one of the primary goals of TKA [1]. Commonly, the value of HKA on full-length films, which formed by the mechanical axis of the tibia and the femur, is used to evaluate the coronal alignment of the lower limb [15, 16]. It has been widely accepted that a HKA within ± 3° deviation from 0° could be regarded as “neutral” [3]. But the technical difficulty to obtain accurate films seemed to hinder the routine use and the necessity of the full-length films decades ago and as a result, when looking back on previous TKA cases, there are circumstances in which the full-length films are not available. As a result, FTA is measured on standing short knee images, and used as a proxy to determine the overall alignment in such circumstances [5]. Our results showed that the consistence between FTA and HKA in assessing coronal alignment was satisfactory.

Commonly, the normal FTA is considered to be around 7° of valgus, and the less FTA value is, the knee become more varus [5]. However, few studies validated accuracy of the FTA criteria using the HKA value, and no consensus has been reached on a certain postoperative FTA to define the neutral alignment and limited the use of short knee film in categorizing the postoperative alignment. Although Fang et al. [5] has claimed an excellent correlation coefficient between the FTA and HKA, with an *r* value more than 0.9, their independent research on this topic cannot be found. To our best knowledge, the only study linking FTA on short films to HKA on full-length films is provided by Park et al., who reported a fair correlation (*r* = 0.69) between FTA and HKA, as well as an unacceptable high error rate (33.0%) when using FTA to categorizing the postoperative alignment [13]. In their study, they used the FTA range proposed by Ritter et al. [10] and regarded the coronalalignment as neutral when the FTA is within 2.4°–7.2° (4.8° ± 2.4°).. However, the general applicability of this criteria is questionable. The mean FTA value is more varus than the widely accepted normal FTA provided by Fang et al. [5]. Based on our data, when classifying the alignment using HKA verses FTA provided by Ritter et al., the concordant rate was only 68.2%, which was very close to that reported by Park et al. [13]. Our results substantiate their conclusion that this FTA range is inadequate to evaluate the coronalalignment. Similar concordant rate, 71.0%, was also acquired when using the FTA provided by Kim et al. [11] However, when using a 4°–9° FTA provided by Morgan et al. [12] to predict the coronal alignment, 82.7% had concordant classifications, which was significantly more accurate than other FTA range reported, and was also slightly more accurate than the FTA calculated in this study. Thus, based on our data, a 4°–9° FTA on short film is the most accurate proxy in predicting the classification of the coronal alignment.

For the coronal alignment of the tibial component in TKA, the prevailing viewpoint is that the tibial component should be positioned perpendicular to the mechanical axis of the tibia to achieve the neutral alignment, representing by a 90° of mMPTA on full-length films. A neutral positioned tibial tray is largely correlated with superior clinical outcomes as well as prolonged implant survivorship after TKA [11]. Both in vitro and in vivo studies have demonstrated that more than 3° varus of tibial tray significantly changed the distribution of the tibial pressure, resulting in higher risk of medial bone collapse, increased insert wear, as well as implant failure [17, 18]. Thus, accurately predicting the alignment of the tibial component is of importance. Although the mechanical axis and the anatomical axis are almost overlapped, whether the aMPTA on short films can accurately predict the mMPTA on full-length films is still not fully validated. According to the results presented by Park et al., when using aMPTA and mMPTA to define the type of the alignment, inconsistent results was obtained in a large amount of patients [13]. Based on our data, the aMPTA on short knee films was marginally more varus than mMPTA on full-length images and the discrepancy was 0.5°. When using the aMPTA to categorize the alignment, the classification of 84.1% of all patients was concordant with that determined by mMPTA on full-length images. Our data substantiated the conclusion that the aMPTA was more varus than the mMPTA, reported by Park et al. [13], however, the discrepancy between the two angles was smaller in our study, and the concordant rate was higher than that in their study.

When using the aLDFA on short films to predict the classification of the alignment of the femoral component, only 57.9% of all patients was concordant with that determined by mLDFA on long images. Unlike the almost coincided mechanical axis and anatomical axis of the tibia, the FAA is of valgus relative to the femoral mechanical axis (FMA), however, the angle between FAA and FMA differs from individual to individual [19]. Although we tried our best to control the postoperative variation by cutting the distal femoral osteotomy in all patients with 6° of valgus relative to the femoral shaft, the concordant rate between aLDFA on short films and mLDFA on long images is still not satisfactory. Nevertheless, in our study, the postoperative FAA was 6.8° of valgus relative to FMA, which was within the 2°–7°safe zone proposed by Gromov et al. [19]. Generally, the short film cannot be a suitable substitute of the full-length film when evaluating the alignment of the femoral component. As a result, the huge discrepancy between the aLDFA and the mLDFA should be the major reason why short knee films cannot accurately predict the overall alignment.

With the application of 3D-CT, the alignment could be easily controlled and measured either during or after TKA procedure [20]. However, the standard weight-bearing knee films and/or the weight-bearing full-length films are still the basic images needed to evaluate the condition both before and after the TKA procedure. This study has several limitations. First, although the system-provided angle-measuring tool minimized the bias produced during the measurement, an obvious limitation is exist when interpreting the results. Second, the rotation of the lower extremity is inevitably different when taking short and long knee films, which could more or less affect the results. In this study, a certain surgeon was sent to help to control the rotation when imaging to minimize the bias caused by the leg rotation, ensuring the accuracy when all analysis were carried out. Third, this study was a single-center study and sample size was limited. Hence, it is essential to confirm our results using patients from other institutions.

## Conclusion

Based on our data, when full-length images are not available and the FTA on standing short knee films is used as a proxy to evaluate the coronal alignment, a 4°–9° FTA is recommended as “neutral alignment”, with more than 80% accuracy. However, if the alignment of the femoral component is to be independently investigated, the full-length film is needed since the short knee films failed to accurately evaluate the alignment of the femoral component.

## Data Availability

The final dataset will be available from the corresponding author.

## Abbreviations

TKA: Total knee arthroplasty
HKA: Hip-knee-ankle angle
FTA: Femorotibial angle
aLDFA: Anatomical lateral distal femoral angle
aMPTA: Anatomical medial proximal tibial angle
mLDFA: Mechanical lateral distal femoral angle
mMPTA: Mechanical medial proximal tibial angle
AP: Anterior–posterior
FAA: Femoral anatomical axis
FMA: Femoral mechanical axis
